# Size Effects in Climatic Aging of Epoxy Basalt Fiber Reinforcement Bar

**DOI:** 10.3390/polym16182550

**Published:** 2024-09-10

**Authors:** Anna A. Gavrilieva, Oleg V. Startsev, Mikhail P. Lebedev, Anatoly S. Krotov, Anatoly K. Kychkin, Irina G. Lukachevskaya

**Affiliations:** 1 Siberian Branch of the Russian Academy of Sciences V.P. Larionov Institute of Physical and Technical Problems of the North, 1 Oktyabrskaya Str., 677000 Yakutsk, Russia; gav-ann@yandex.ru (A.A.G.); startsevov@gmail.com (O.V.S.); askrotov@list.ru (A.S.K.); mirkin1611@gmail.com (I.G.L.); 2 Siberian Branch of the Russian Academy of Sciences Federal Research Center «Yakut Scientific Center SB RAS», 2 Petrovskogo Str., 677000 Yakutsk, Russia; m.p.lebedev@mail.ru

**Keywords:** basalt-fiber-reinforced polymer (BFRP), anisotropy, size effect, climatic aging, Langmuir model, glass transition temperature, coefficient of linear thermal expansion, strength, post-curing

## Abstract

The purpose of this study was to obtain information on the influence of the size factor on the climatic aging of circular fiber plastics produced by pultrusion. The kinetics of moisture transfer was obtained in humidification and drying modes at 60 °C in samples of epoxy basalt fiber reinforcement bars: after 28 months of exposure in the extremely cold climate of Yakutsk and 30 months of exposure in the moderately warm climate of Gelendzhik. It was shown that the 2D Langmuir model adequately describes the kinetics. The diffusion coefficients in the reinforcement direction for bars with diameters of 6, 8, 10, 16 and 20 mm turned out to be significantly higher than in the radial direction. To clarify the aging mechanism of the bars and the tensile, compressive and bending strength, the coefficient of linear thermal expansion and the glass transition temperature of the epoxy matrix of the bars with a diameter of 6, 8 and 10 mm after 51 months of exposure in Yakutsk and 54 months of exposure in Gelendzhik were measured. It was shown that after climatic exposure, the deformability of the bars decreased with increasing diameter of the bar; the glass transition temperature increased more significantly in the bar with a smaller diameter. In 6 mm diameter bars, the compressive and bending strength limits decreased by 10–25 % due to the plasticizing effect of moisture. With the same depth of moisture penetration into the volume of the samples, its effect on the strength of thin bars was significant, and for thick bars, it was insignificant. An increase in the glass transition temperature by 6 °C, associated with the additional curing of the polymer matrix, occurred in the surface layer of the epoxy basalt fiber reinforcement bars and was revealed in bars with a smaller diameter.

## 1. Introduction

Basalt fiber is used to manufacture fire-resistant heat and sound insulating systems [1], but their main purpose is associated with the creation of basalt-fiber-reinforced polymers (BFRPs). Reviews [1,2,3,4,5,6,7,8,9] have reported on the manufacture of various building elements from BFRP. The choice of basalt fiber as a fibrous reinforcing component among various FRPs is based on its positive qualities such as high strength, heat resistance, resistance to chemically active compounds [3,6,7,8,9,10], fire resistance, inertness to mold and microorganisms [1] and resistance to abrasion and impact loads [4]. According to [2], basalt fiber has higher levels of adhesive interaction with epoxy, phenolic and other polymer matrices than fiberglass.

Experimental studies have proven the advantages of basalt fiber reinforcement plastic (BFRP) over fiberglass in terms of strength [3,11,12,13,14,15,16], fatigue [17], durability [18,19] and resistance to chemically active environments [20]. An analysis of existing data on the key characteristics of glass–basalt fiber reinforcement plastics, such as high tensile strength, exceptional flexural strength, low thermal conductivity, favorable density, chemical resistance, impact resistance, fire resistance and general weather resistance, have revealed the potential for the use of these materials in construction in temperate and arctic climates [21].

A characteristic feature of FRPs is the dependence of their resistance parameters *R* (tensile $\sigma_{t}$, strength $\sigma_{c}$ and bending $\sigma_{b}$ strength) on the shape and size of the samples [22,23,24,25,26,27]. For example, according to the work [25], the $\sigma_{b}$ indicator in a unidirectional fiberglass rod with a diameter of 9.5 mm was 1620 MPa, and with an increase in diameter to 25.4 mm, it had a value of 1410 MPa (decreased by 15%).

Differences in *R* values can be caused not only by the form factor but also by unequal properties of the initial components [28], the influence of curing modes [29], the occurrence of gradients in thickness values [30] and the influence of test rig when performing mechanical measurements [31,32,33]. In each specific case, identifying these reasons is a prerequisite for a reasonable interpretation of the results of climate tests and requires additional labor-intensive research.

The scientific literature contains many examples showing that, after the same exposure to an aggressive external environment, the aging effects of FRPs significantly depend on the shape and size of the tested samples. The authors of [34] studied the thermal aging of unidirectional epoxy carbon-fiber-reinforced plastic at 175 °C for 250 h and compared the properties of four- and eight-layer plates. For four-layer plates, the $\sigma_{t}$ in the 90 direction index decreased from 52.2 to 44.7 MPa (by 14%). In the case of the eight-layer plate, a similar decrease occurred from 48.0 to 23.6 MPa (by 51%).

According to [35], the $\sigma_{c}$ index for carbon fiber plastic with a thickness of 26 mm (100 layers) after 30,000 h of thermal cycling from −50 to 150 °C decreased by 25%, and in thin samples (5 mm, 20 layers), the decrease was 50%. This significant difference is due to the dominant influence of the oxidation of surface layers, as a result of which the rate of decrease in strength of thin samples increased by 2.4 times.

Fiberglass with unidirectional fibers, fabric and randomly arranged chopped strands with a thickness of 2 and 5 mm was tested for 2000 h under thermal cycles from −20 to 20 °C in dry air, when immersed in water and with combined exposure to moisture and UV radiation [23]. In all exposure options, the decrease in the $\sigma_{t}$ index of thin samples was more significant. For example, in fiberglass with chopped fiber during UV cycling, $\sigma_{t}$ decreased by 26% in samples with a thickness of 5 mm and by 42% in samples with a thickness of 2 mm.

The effect of water as an aggressive environmental factor was discussed in [36]. The dependence of the tensile strength on the thickness of samples of unidirectional epoxy basalt plastic was revealed. After 180 days of exposure in water at a temperature of 60 °C, the $\sigma_{t}$ index of samples with a thickness of 1, 2 and 4 mm decreased, respectively, by 66, 62 and 59%.

In the examples considered, temperature, humidity and UV radiation caused the destruction of C-O bonds, the formation of voids and microcracks mainly in the surface layers of FRPs [23,34,35,36]. The formation of a gradient of *R* values across thickness during climatic aging [28] is the main reason for the emerging differences in the properties of FRPs of different thicknesses. Therefore, when conducting comparative climatic tests of FRPs, it is advisable to use plates with the same reinforcement patterns and thickness or use rods with the same reinforcement patterns and equal diameters, manufactured using identical pressing conditions from identical semi-finished products.

The formation of a gradient of *R* values in thickness during climatic aging fully applies to fiber-reinforced plastics with a round cross-section, manufactured by pultrusion. Thus, it is necessary to take into account the unidirectional anisotropy of moisture transfer (differences in diffusion coefficients in the direction of reinforcement and in the radial direction) in the problem of assessing the climatic aging of FRPs by changing the parameters of moisture transfer under stationary thermal and humidity conditions [37,38,39]. Previously, the work [40] reported differences in diffusion coefficients and moisture content in BFRP bars depending on their diameter after exposure to extremely cold climates, but to approximate the identified differences, Fick’s second law was used in a one-dimensional approximation. Moreover, numerous studies show that moisture absorption in FRPs is pseudo-anomalous. It is characterized by Fickian kinetics at the initial stage of the process and a very fast or very slow final stage of establishing equilibrium. To adequately determine diffusion coefficients, it is necessary to separate the pseudo-equilibrium moisture content from the total equilibrium moisture content. The well-known Langmuir model, which is described in works [41,42,43], is suitable for this purpose.

In the moisture desorption mode, moisture transfer makes it possible to assess the state of the polymer matrix in aged FRPs without additional physicochemical transformations. In the absorption mode, especially at elevated temperatures, water molecules activate the processes of hydrolysis and post-curing [44]. Therefore, it is advisable to carry out the kinetics of moisture transfer in FRPs after climatic aging in the modes of moisture absorption–desorption, especially for rods of different diameters.

Thus, the purpose of this work is to obtain additional information about the influence of the size factor on the climatic aging of BFRP bars. For this purpose, the analysis of moisture transfer by the 2D Langmuir model was supplemented by a study of the strength and physical characteristics of BFRP bars of different diameters after 51–54 months of exposure in two climatic zones.

## 2. Materials and Methods

### 2.1. Materials

The object of this study is a BFRP bar—unidirectional reinforced rods with a periodic profile (TBM LLC, Yakutsk, Russia) with a high volume content of basalt reinforcing fibers (0.79 wt.%). The reinforcing filler is basalt roving RBN 13-2400-4S (TBM LLC, Yakutsk, Russia). The epoxy binder consists of ED-22 epoxy resin cured with iso-MTHFA isomethyltetrahydrophthalic anhydride in the presence of an accelerator 2,4,6-tris (dimethylaminomethyl) phenol UP-606/2 (100:75:1.3). Performance parameters are shown in Table 1.

### 2.2. Specimen Preparation

The BFRP bar was manufactured on the “Struna” technical line in accordance with Technical Specifications 2296-001-86166796-2013 “Non-metallic composite reinforcement made of basalt plastic”. The line consists of the following stages: impregnation of basalt roving with a binder; formation of a power central rod: drawing the rovings through the central hole of the drawing die; formation of the outer layer of the rod: applying the rovings through the side holes of the drawing die onto the power central rod; formation of a periodic layer of the bar: winding the resulting rod of rovings with polyamide thread; bar curing.

Technological modes for forming bars are presented in Table 2.

### 2.3. Climate Aging Test

The exposure of the BFRP bars was carried out in accordance with GOST 9.708-83 [45] in the extremely cold climate of Yakutsk and in the moderately warm climate of Gelendzhik. The samples were exposed to natural climatic factors in a free state. Average annual climatic characteristics of Yakutsk and Gelendzhik are presented in Table 3.

### 2.4. Aging Parameter Determination Methods

#### 2.4.1. Coefficient of Linear Thermal Expansion (CLTE)

The CLTE of BFRP bars in the direction of the reinforcing basalt fibers was tested using a Thermomechanical analyzer TMA 202 (Netzsch-Geratäbau GmbH, Selb, Germany) in accordance with GOST 326182-2014 [46]. Samples were heated from −80 °C to 200 °C at a rate 5 °C/min. The load on the sample was 0.03 N. To increase the reliability of the results, 5 parallel samples measuring 20 × 5 × 5 mm were used. The ends of the samples were faced on a lathe until the deviation from plane-parallelism was no more than 0.01 mm.

#### 2.4.2. The Glass Transition Temperature ($T_{g}$)

The $T_{g}$ and the transition boundaries of epoxy binder from the glassy to highly elastic state were determined with an accuracy of 0.5 °C according to the recommendations of [29]. The dynamic storage modulus $E^{\prime }$ and the dynamic loss modulus $E^{\prime \prime }$ were measured using a Dynamic Mechanical Analysis DMA 861 (Netzsch-Geratäbau GmbH, Germany) in accordance with GOST R577399-2017 [47] during bending vibrations of a 50 mm long block cut from BFRP. In each measurement cycle, 3 parallel samples were used. Measurements were carried out in the temperature range from −10 to 150 °C at a heating rate of 5 K/min and a frequency of 1 Hz.

#### 2.4.3. Mechanical Parameters ($\sigma_{t}$, $\sigma_{b}$, $\sigma_{c}$)

Measurements of tensile, compressive strengths in accordance with GOST 32492-2015 [48] and bending (flexural) strengths in accordance with ASTM D790-17 [49] were carried out on a Z100 Zwick/Roell (ZwickRoell GmbH, Ulm, Germany) testing machine at room temperature. Loading was carried out until the samples failed in the working area. To reduce the influence of scatter, 10 parallel samples were used in each batch of measurements with a length of 80 mm and with a nominal diameters 6, 8 and 10 mm.

#### 2.4.4. Moisture Transfer Parameters ($D_{r}$, $D_{h}$, γ, β)

A cylinder with radius *R* and height 2h is considered in which there are $c_{m}$ mobile water molecules which diffuse with 2D diffusion coefficient ($D_{r}$, $D_{z}$) and become bound at a rate $\gamma c_{m}$. At the same time, there are $c_{b}$ bound molecules which become mobile at a rate $\beta c_{b}$. Concentrations $c_{m}$, $c_{b}$ are then a function of radius *r*, height *z* and time *t* only. The diffusion Fick’s equation [50] and the Langmuir equation of adsorption [51] become

(1) $$\begin{array}{c} \frac{𝜕c_{m}\left(r,z,t\right)}{𝜕t}+\frac{𝜕c_{b}\left(r,z,t\right)}{𝜕t}=D_{r}\left(\frac{{𝜕}^{2}c_{m}\left(r,z,t\right)}{𝜕r^{2}}+\frac{1}{r}\frac{𝜕c_{m}\left(r,z,t\right)}{𝜕r}\right)+D_{z}\frac{{𝜕}^{2}c_{m}\left(r,z,t\right)}{𝜕z^{2}},\\ \frac{𝜕c_{b}\left(r,z,t\right)}{𝜕t}=\gamma c_{m}-\beta c_{b};\\ 0\leq r\leq R,\;-h\leq z\leq h,\;t\geq 0. \end{array}$$

In the initially dry cylinder exposed to a constant moisture environment, the initial and boundary conditions are

(2) $$\begin{array}{c} c\left(r,z,0\right)=c_{m}\left(r,z,0\right)+c_{b}\left(r,z,0\right)=0,\\ c(R,z,t)=c(r,-h,t)=c(r,h,t)=const,\;\\ c(0,z,t)<\infty . \end{array}$$

When both γ and β are small compared to the rate of saturation of a cylinder $\frac{\pi^{2}D_{h}}{{\left(2h\right)}^{2}}$, $\frac{J_{0}\left(1\right)D_{r}}{R^{2}}$, then, the integration of the above results (1) and (2) over volume gives the moisture content in an initially dry cylinder, after exposure time *t*, which is given approximately by [51,52]

(3) $$\begin{array}{c} M\left(t\right)=M_{0}\left(1-\frac{\gamma }{\gamma +\beta }\exp\left(-\beta t\right)-\frac{\beta }{\gamma +\beta }\sum_{n=1}^{\infty }\frac{4}{\mu_{n}^{2}}e^{-\mu_{n}^{2}\frac{D_{r}}{R^{2}}t}\sum_{m=1}^{\infty }\frac{2}{\mu_{m}^{2}}e^{-\mu_{m}^{2}\frac{D_{z}}{h^{2}}t}\right);\\ \gamma ,\;\beta <\frac{\pi^{2}D_{h}}{{\left(2h\right)}^{2}};\;\frac{J_{0}\left(1\right)D_{r}}{R^{2}}, \end{array}$$

where $M_{0}$ is a moisture equilibrium content, $J_{0}\left(\mu_{n}\right)=0$ are zeros of the Bessel function of the 1st kind of zero order, $\mu_{m}=\left(2m-1\right)\pi /2$.

Here and below, the superscript ^d^ means that the corresponding value refers to the process of moisture desorption. To determine the moisture content during subsequent desorption $M^{d}\left(t\right)$, it is necessary to solve Equation (1) with the initial concentration value equal to the concentration distribution at the last moment of absorption $t_{s}$ and zero boundary conditions

(4) $$\begin{array}{c} c^{d}\left(r,z,t_{s}\right)=c_{m}\left(r,z,t_{s}\right)+c_{b}\left(r,z,t_{s}\right),\\ c^{d}\left(R,z,t\right)=c^{d}\left(r,-h,t\right)=c^{d}\left(r,h,t\right)=0,\;\\ c^{d}\left(0,z,t\right)<\infty . \end{array}$$

The integration of the above results (1) and (4) over volume gives the total moisture content after desorption time *t*, which is given approximately by

(5) $$\begin{array}{c} M^{d}\left(t+t_{s}\right)=M\left(t_{s}\right)-\\ -M_{0}^{d}\left(1-\frac{\gamma^{d}}{\gamma^{d}+\beta^{d}}\exp\left(-\beta^{d}t\right)-\frac{\beta^{d}}{\gamma^{d}+\beta^{d}}\sum_{n=1}^{\infty }\frac{4}{\mu_{n}^{2}}e^{-\mu_{n}^{2}\frac{D_{r}^{d}}{R^{2}}t}\sum_{m=1}^{\infty }\frac{2}{\mu_{m}^{2}}e^{-\mu_{m}^{2}\frac{D_{z}^{d}}{h^{2}}t}\right);\\ \gamma^{d},\;\beta^{d}<\frac{\pi^{2}D_{h}^{d}}{{\left(2h\right)}^{2}};\;\frac{J_{0}\left(1\right)D_{r}^{d}}{R^{2}}. \end{array}$$

The validity of the 2D Langmuir models (3) and (5) can be determined by gravimetric experiments of the water absorption and desorption of a BFRP bar that is initially dry and then exposed to conditions of fixed humidity and temperature.

#### 2.4.5. Kinetics of Absorption–Desorption

**Figure 1:**
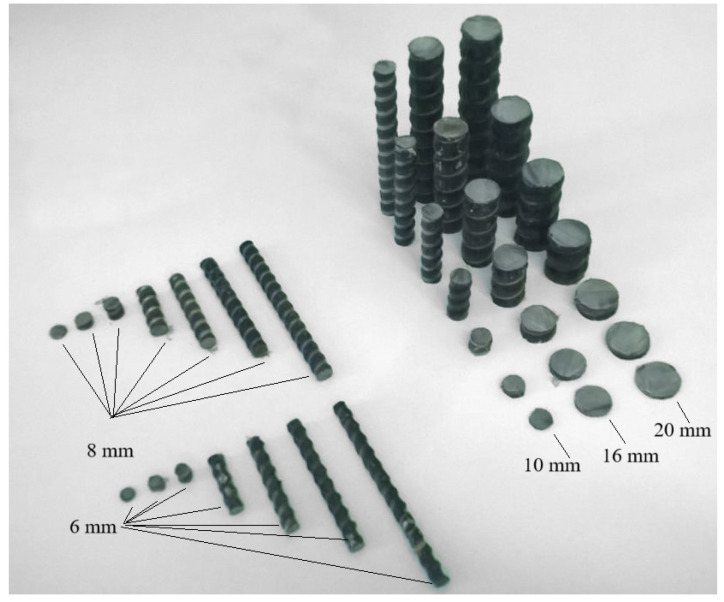
A set of BFRP bar samples with a nominal diameter 6, 8, 10, 16 and 20 mm to determine moisture transfer characteristics.

From BFRP bars unexposed and exposed to climatic conditions with a nominal diameter of 6, 8, 10, 16 and 20, five samples of length 10, 30, 50, 70 and 100 mm were cut (Figure 1).

The experimental values of the kinetics of moisture sorption and desorption in BPA were determined according to ASTM D5229/D5229M-20 [53]. The specimens were pre-dried in a heating oven at a temperature of 60 °C over silica gel for 6 days to remove the water absorbed. The mass was weighed and recorded as $m_{0}$. After drying, the specimens were kept at the same temperature and a relative humidity of 98 ± 2 % in a desiccator above the water surface. At time *t*, the mass of the specimens was measured on an analytical balance with an accuracy of 0.0001 g, being recorded as $m_{t}$. Then, the moisture absorption process was stopped, and the samples were dried in a heating oven at a temperature of 60 °C. The moisture content in a specimen at time *t*, $M_{t}$ (%) was calculated by

(6) $$M_{t}=\frac{m_{t}-m_{0}}{m_{0}}\cdot 100.$$

## 3. Results and Discussion

### 3.1. Size Effect in the Deformability of Exposed BFRP Bars

**Figure 2:**
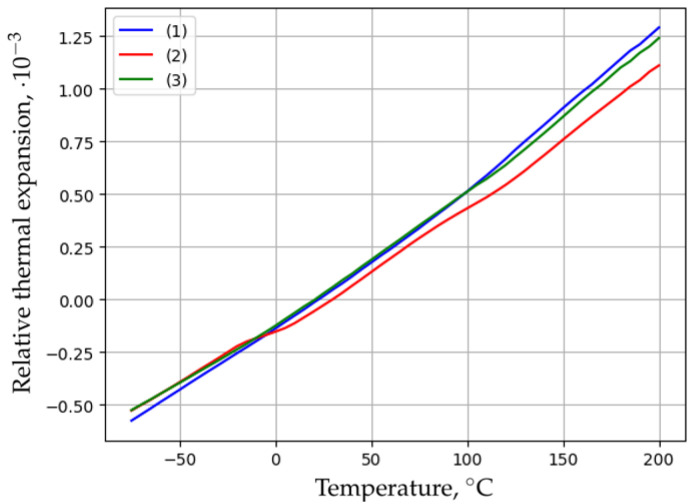
Temperature dependences of the relative thermal expansion of three parallel BFRP 6 samples after 54 months of exposure in Yakutsk.

A typical example of the thermal expansion of the BFRP bar is shown in Figure 2. This figure shows the temperature dependence of the relative elongation of three parallel samples of BFRP 6 after 54 months of exposure in Yakutsk.

**Figure 3:**
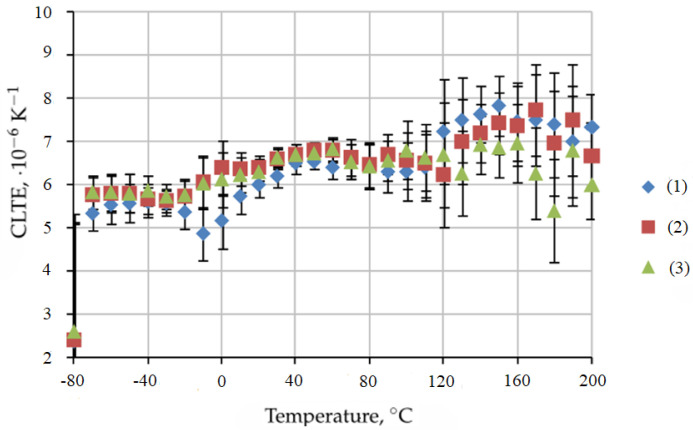
Average CLTE values of BFRP bar samples after 54 months of exposure in Yakutsk: (1) BFRP 6; (2) BFRP 8; (3) BFRP 10.

The obtained dependencies are well reproduced, and the scatters are insignificant. This makes it possible to determine the CLTE with good accuracy over a wide temperature range. The average values of CLTE of the BFRP bar samples after 54 months of exposure in Yakutsk are presented in Figure 3.

The temperature range from −60 °C to 110 °C corresponds to the glassy state of the epoxy matrix. Within this interval, as the temperature increases, the CLTE of BFRP 6 increases insignificantly from $5.5\cdot 10^{-6}$ $K^{-1}$ to $6.4\cdot 10^{-6}$ $K^{-1}$. Above a temperature of 110 °C, the CLTE increases to $7.8\cdot 10^{-6}$ $K^{-1}$ due to the appearance of segmental mobility and the transition of the binder to a highly elastic state. For BFRP 10, the CLTE in the specified temperature range varies within smaller limits (from $5.8\cdot 10^{-6}$ $K^{-1}$ at −60 °C to $6.0\cdot 10^{-6}$ $K^{-1}$ at 200 °C with a maximum of $6.9\cdot 10^{-6}$ $K^{-1}$ at 140 °C). The corresponding CLTE values for BFRP 8 occupy an intermediate position (Figure 3).

Thus, the deformability of the BFRP bar exposed in Yakutsk at elevated temperatures decreases with increasing bar diameter.

### 3.2. Size Effect in the Post-Curing of Exposed BFRP Bars

In the work [29], the options for finding the glass transition temperature $T_{g}$ of epoxy polymers and FRPs based on them are considered in detail at the beginning and end of the intersection, at the middle of the interval, the inflection point on the graphs of the dynamic shear moduli $G^{\prime }$, $G^{\prime \prime }$ and tanδ on the graphs of the dynamic elastic moduli $E^{\prime }$, $E^{\prime \prime }$ and tanδ. The recommendations of ASTM E1356-08 [54], ASTM-E1640 [55] and ISO 11357-2 [56], ISO-6721-11 [57] standards for DSC and DMA measurements of epoxy polymers are considered in detail. A conclusion is made about the equivalence of $T_{g}$ determined by the midpoint of the heat flux jump, the temperature of the $G^{\prime \prime }$ or $E^{\prime \prime }$ peaks and the temperature of the minimum of the temperature derivative $dE^{\prime \prime }/dT\left(T\right)$.

The authors of the work recommend using the DMA method to find $T_{g}$ both from the position of the maximum of $G^{\prime \prime }$ and from the position of the maximum of tanδ. However, as shown in [29], when comparing the DSC and DMA methods, $T_{g}$ determined from the position of the maximum of tanδ is always significantly higher than that determined from the DSC data.

Thus, determining $T_{g}$ based on the position of the maximum $E^{\prime \prime }$ gives a more reliable value of the glass transition temperature, which coincides with the DSC data. This method of determination is used in works [58,59,60,61].

**Figure 4:**
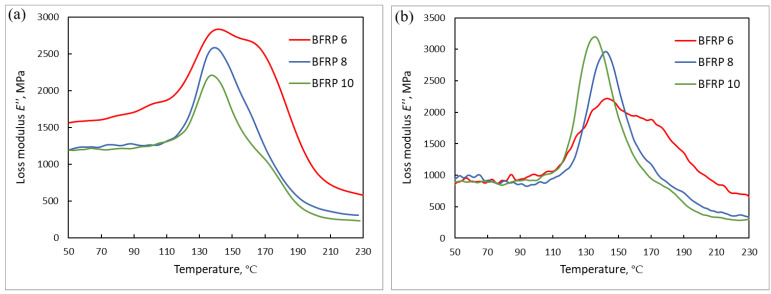
(**a**) A typical example of temperature dependences of the dynamic loss modulus of BFRP samples after 54 month of exposure in Yakutsk; (**b**) A typical example of temperature dependences of the dynamic loss modulus of BFRP samples after 64 month of exposure in Gelendzhik.

Figure 4 shows the dependence of the dynamic loss modulus on temperature $E^{\prime \prime }\left(T\right)$ of BFRP samples after exposure in Yakutsk and Gelendzhik.

The measured dependences $E^{\prime \prime }\left(T\right)$ made it possible to determine with sufficient accuracy the glass transition temperature $T_{g}\left(E_{max}^{\prime \prime }\right)$ (from the position of the maximum $E^{\prime \prime }\left(T\right)$), the lower $T_{i}$ and upper $T_{f}$ boundaries of the transition of the BFRP epoxy matrix from the glassy to the highly elastic state [29,37]. The values of these characteristic temperatures are presented in Table 4.

The glass transition temperature $T_{g}\left(E_{max}^{\prime \prime }\right)$ of an epoxy BFRP 6 matrix increased by 6 °C after exposure in Yakutsk and by 10 °C after exposure in Gelendzhik. The effect decreased as the bar diameter increased. For bars with a nominal diameter of 10 mm, the increase in $T_{g}\left(E_{max}^{\prime \prime }\right)$ did not exceed 1–2 °C. An increase in $T_{g}$ is a sign of the post-curing of the epoxy matrix [37]. In [28], it was shown that post-curing is activated under conditions of elevated levels of temperature and relative air humidity, that is, in the climate of Gelendzhik. Since during the exposure time, moisture manages to penetrate only into the surface layer of the BFRP bars, the main volume of the large-diameter bars remained inaccessible to water molecules, which did not lead to an increase in $T_{g}$.

### 3.3. Size Effect in the Strength of Exposed BFRP Bars

The size effect of the climatic aging of BFRP bars was also revealed by the results of measuring the strength indicators of the bars (Table 5); the standard deviation of the tensile strength values did not exceed 3 %, and the bending and compressive strength values did not exceed 2 %.

It turned out that the tensile strength $\sigma_{t}$, compression $\sigma_{c}$ and bending $\sigma_{b}$, measured after 28–30 months and 51–54 months of exposure, increased by 5–11% in BFRP 10. A similar increase in $\sigma_{t}$ to 14% occurred in a BFRP 6. Based on the results of TMA and DMA (Figure 4, Table 4), it can be argued that the increase in $\sigma_{t}$ is due to post-curing and an increase in the rigidity of the binder in the direction of reinforcement. At the same time, the indicators $\sigma_{c}$ and $\sigma_{b}$ decreased by 10–25% in thin bars (Table 5). This discrepancy can be explained by the plasticizing effect of moisture. At the same depth of moisture penetration, its effect on the strength of thin bars turned out to be significant, but for thick bars, it was insignificant.

Therefore, moisture diffusion is one of the main reasons for changes in *R* values during the climatic exposure of BFRP bars. Modeling moisture transfer helps to understand and substantiate the causes of the observed size effect in the aging.

### 3.4. Size Effect in the Moisture Transfer Parameters of Exposed BFRP Bars

**Figure 5:**
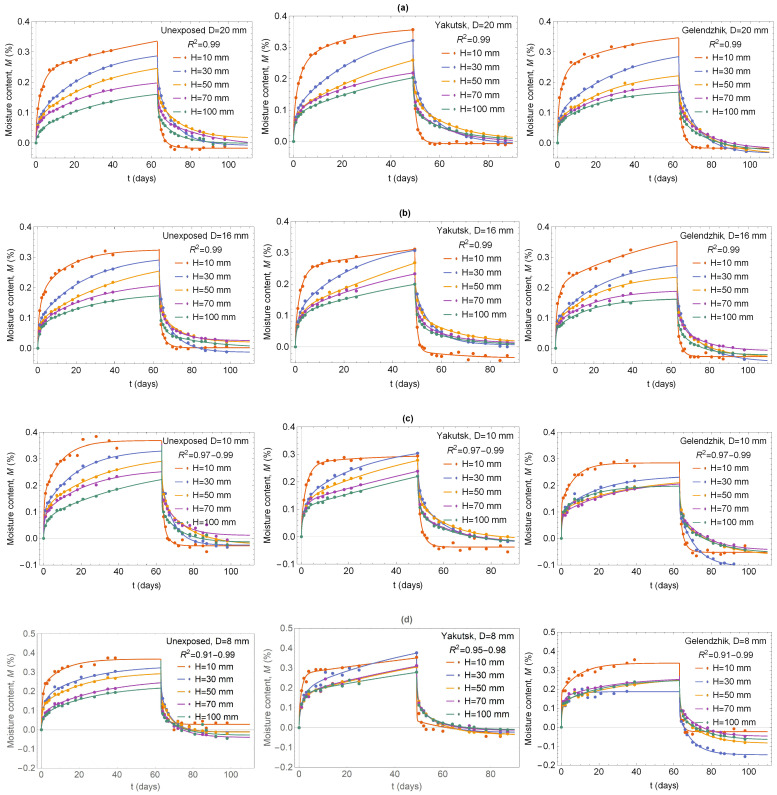

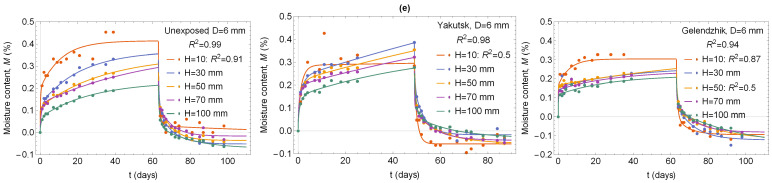
Kinetic curves of the absorption–desorption cycle of BFRP bars samples unexposed, after 28 months of exposure in Yakutsk and after 30 months of exposure in Gelendzhic at RH 98% and 60 °C: (**a**) D = 20 mm, (**b**) D = 16 mm, (**c**) D = 10 mm, (**d**) D = 8 mm, (**e**) D = 6 mm. Solid curves from Equations (3) and (5).

The 2D Langmuir model (3) and (5) was used to best fit the data of absorption and desorption cycle in initially dry BFRP bar samples by a least-squares fit. In the formulas (3) and (5), ten terms were held in the infinite sum, which is enough for a satisfactory estimate. The fit was made using a Walfram Mathematica (free 15-day trial of Mathematica, https://www.wolfram.com/mathematica/trial/, accessed on 1 March 2024). A portion of samples were unexposed (they were stored in a warehouse) and a portion of samples were exposed to the climatic conditions of Yakutsk for 28 months or Gelendzhik for 30 months: with nominal diameter 6, 8, 10, 16 and 20 and length 10, 30, 50, 70 and 100 mm. The results of fits by the 2D Langmuir model, depicted in Figure 5, show good adequacy of the model (determination coefficient $R^{2}=0.91-0.99$) and a description of the absorption–desorption curves with an account of the two-phase nature of moisture and the two components of diffusion.

**Figure 6:**
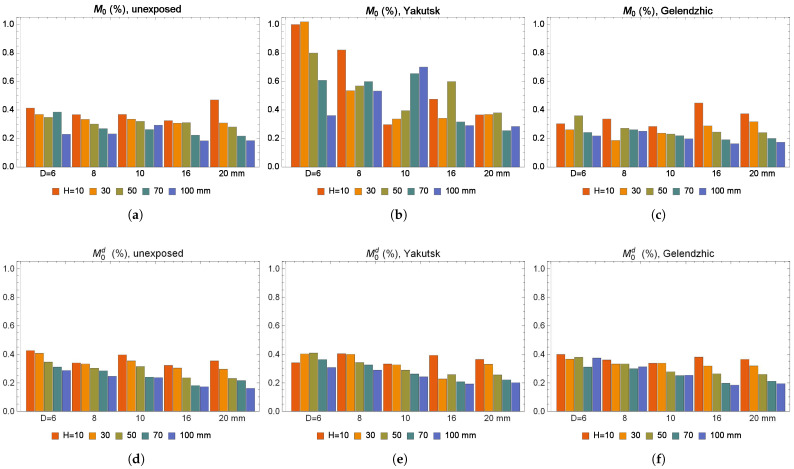
Moisture equilibrium content during absorption of BFRP bar samples that are (**a**) not exposed, (**b**) exposed in Yakutsk for 28 months, (**c**) exposed in Gelendzhik for 30 months. Moisture equilibrium content during desorption of BFRP bar samples that are (**d**) not exposed, (**e**) exposed in Yakutsk for 28 months, (**f**) exposed in Gelendzhik for 30 months.

The results of approximation for absorption using Formula (3) and desorption using Formula (5) for the equilibrium moisture contents $M_{0}$ and $M_{0}^{d}$ of the BFRP bar samples are shown in Figure 6, except for samples BFRP 6 and 8 after exposure in Yakutsk, for which the value $M_{0}$ does not exceed 1%. It can be seen that the BFRP bar samples absorb a relatively small amount of moisture < 0.4%, whereas the epoxy binder absorbs a relative amount of moisture of about 3% [50,62], and the glass-fiber-reinforced epoxy resin absorbs a relative amount of moisture of about 1% [41]. There is an increase in the value $M_{0}$ and $M_{0}^{d}$ for BFRP samples with decreasing sample height.

**Figure 7:**
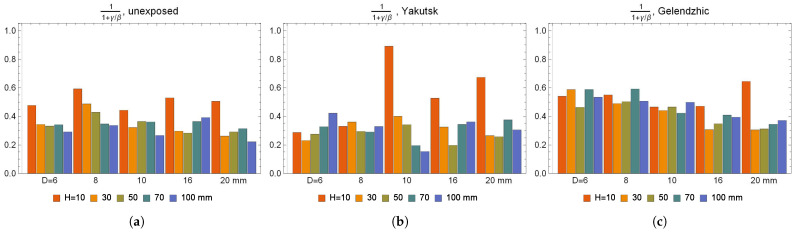
The proportion of pseudo-equilibrium in the total equilibrium content of moisture during absorption of BFRP bar samples that are (**a**) not exposed, (**b**) exposed in Yakutsk for 28 months, (**c**) exposed in Gelendzhik for 30 months.

The results of approximation for absorption using formula (3) and for the probability that a mobile water molecule becomes bound γ and probability that a bound water molecule becomes mobile β of the BFRP bar samples are shown in Figure 7. For samples unexposed and exposed in Yakutsk, we can conclude that $\frac{1}{1+\gamma /\beta }$, which corresponds to the proportion of pseudo-equilibrium in the total equilibrium content of moisture [63], was constant and amounted to about 0.3. A similar proportion was observed for the epoxy binder, but the values of γ and β were ten times lower [62], whereas for BFRP 6 and BFRP 8 exposed in Gelendzhik, the proportion was about 0.5, and for BFRP 16 and BFRP 20 exposed in Gelendzhik, the proportion was also about 0.3.

For BFRP 6 and BFRP 8 exposed in Gelendzhik, the values of γ are approximately equal to the value of β, which means that the probabilities that a mobile water molecule becomes bound and vice versa are equal.

**Figure 8:**
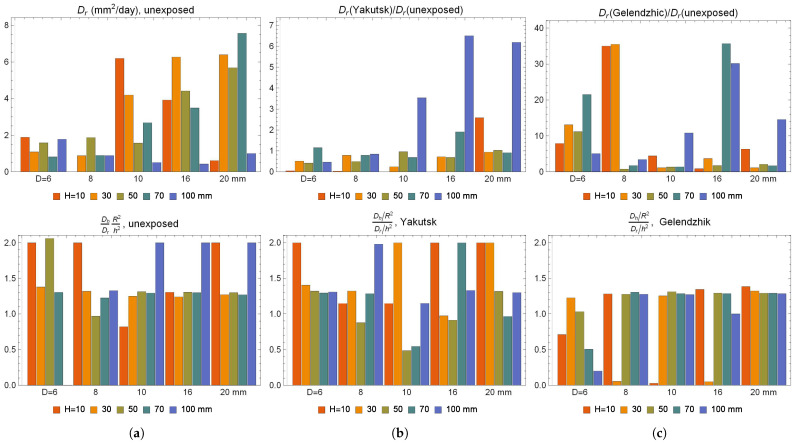
Comparative analysis of diffusion coefficients during absorption of BFRP bar samples that are (**a**) not exposed, (**b**) exposed in Yakutsk for 28 months, (**c**) exposed in Gelendzhik for 30 months.

And finally, the comparative analysis of the approximation for absorption using Formula (3) and desorption using Formula (5) for the radial component of the diffusion coefficient $D_{r}$ of the BFRP bar samples are shown in Figure 8. The $D_{r}$ for BFRP 6 exposed in Gelendzhik increased significantly by about 10 times compared to $D_{r}$ of unexposed BFRP 6. Moreover, the ratio of the radial diffusion Fourier number $Fo_{r}$ to the longitudinal diffusion Fourier number: $Fo_{h}$

$$\frac{Fo_{r}}{Fo_{h}}\equiv \frac{D_{r}/R^{2}}{D_{h}/h^{2}}\approx 1.3,$$

was approximately 1.3, whereas for BFRP 6 exposed in Gelendzhik, this ratio depended on the height of the sample.

## 4. Conclusions

This study showed that the effects of climatic aging of BFRP bars depend on their diameter and the climatic conditions of exposure. The deformability of the BFRP bars exposed in Yakutsk at elevated temperatures decreases with increasing rod diameter. An increase in the glass transition temperature associated with the post-hardening of the polymer matrix occurs in the surface layer and is detected in bars with a smaller diameter. At the same depth of atmospheric moisture penetration into the volume of the samples, its effect on the strength of thin rods turns out to be significant, but for thick bars, it is insignificant.

The diffusion of atmospheric moisture is one of the main reasons for changes in strength indicators during the climatic exposure of BFRP bars. The adequacy of the 2D Langmuir model for the cycle of sorption and desorption of moisture in the BFRP bar for samples of different sizes at a thermal humidity regime of 60 °C and a relative humidity of 98% has been shown. The diffusion coefficients in the reinforcement direction for bars of all studied diameters are higher than in the radial direction. The ratio of the radial diffusion Fourier number to the diffusion Fourier number along the reinforcement $\frac{Fo_{r}}{Fo_{h}}$ parameter well describes the size effect of the climatic aging of FRPs due to the absorption of atmospheric moisture.

## Figures and Tables

**Table 1:** Performance parameters of components.

ED-22		iso-MTHFA		UP-606/2		RBN 13-2400-4S	
Epoxy group (wt. %)	21.4	Main substance (wt.%)	99.2	Main substance (wt.%)	98.4	Linear density (tex)	2400
Viscosity at 25 °C (Pa·sec)	16	Viscosity at 20 °C (sec)	28	Density at 25 °C (kg/m)^3^	10	Breaking load (mN/tex)	46
Appearance	Transparent	Appearance	Transparent	Appearance	yellow	fiber diameter (μm)	13

**Table 2:** Technological modes for forming BFRP bars.

Nominal Bar Diameter ^1^ (mm)	Central Rod Diameter (mm)	Tension of Polyamide Winding Threads (kg)	Polymerizer Temperature (°C)	Tensile Strength (MPa) ^2^	Tensile Modulus (GPa) ^2^
6	4	3	125	1260	53
8	6	4	125	1220	52
10	8	6	125	1210	52
16	13	6	130	1199	51
20	17	8	140	1190	50

^1^ used when calculating the mechanical characteristics of bar, the diameter of the bar without a periodic profile. ^2^ according to the test protocols of TBM LLC.

**Table 3:** Average annual climatic characteristics of Yakutsk and Gelendzhik.

Impact Factor	Yakutsk	Gelendzhik
Relative humidity (%)	68	73
Wind speed (m/s)	1.8	3.5
Average maximum air temperature (°C)	−3.4	24.8
Average minimum air temperature (°C)	−14.1	5
Average air temperature (°C)	−8.8	14.8
Precipitation rate (mm)	237	665

**Table 4:** Size effect in the glass transition temperature of the BFRP bar epoxy matrix.

Climate Aging Test	Nominal Bar Diameter (mm)	$T_{i}{(}^{{}^{\circ}}C)$	$T_{g}\left(E_{\max}^{\prime \prime }\right){(}^{{}^{\circ}}C)$	$T_{f}{(}^{{}^{\circ}}C)$
unexposed	6	104 ± 1	133 ± 1	190 ± 1
	8	105 ± 1	133 ± 1	190 ± 1
	10	104 ± 1	134 ± 1	190 ± 1
exposed for 51 month in Yakutsk	6	110 ± 2	139 ± 1	200 ± 2
	8	107 ± 2	137 ± 1	200 ± 2
	10	105 ± 2	135 ± 1	200 ± 2
exposed for 54 month in Gelendzhik	6	110 ± 2	143 ± 1	200 ± 2
	8	110 ± 2	140 ± 1	200 ± 2
	10	106 ± 2	136 ± 1	200 ± 2

**Table 5:** Size effect in the strength indicators of BFRP bars.

Climate Aging Test	Nominal Bar Diameter (mm)	Tensile Strength (MPa)	Bending Strength (MPa)	Compressive Strength (MPa)
unexposed	6	1120 (1.0) ^1^	1209 (1.0)	410 (1.0)
	8	1003 (1.0)	764 (1.0)	466 (1.0)
	10	-	624 (1.0)	432 (1.0)
exposed for 28 months in Yakutsk	6	1206 (1.0)	1087 (0.90)	428 (1.04)
	8	1078 (1.07)	733 (0.96)	474 (1.02)
	10	-	639 (1.02)	452 (1.05)
exposed for 51 months in Yakutsk	6	1275 (1.14)	976 (0.80)	-
	8	1080 (1.03)	666 (0.87)	-
	10	-	695 (1.11)	-
exposed for 30 months in Gelendzhik	6	-	1094 (0.90)	427 (1.04)
	8	-	658 (0.86)	420 (0.90)
	10	-	612 (0.98)	454 (1.05)
exposed for 54 months in Gelendzhik	6	1193 (1.07)	920 (0.76)	-
	8	1029 (1.03)	730 (0.96)	-
	10	-	677 (1.08)	-

^1^ the values of the conservation coefficients $k=R/R_{0}$ are indicated in parentheses, where *R* is the strength indicator measured for exposed sample, $R_{0}$ is the value of the indicator measured for unexposed sample.

## Data Availability

The original contributions presented in the study are included in the article, further inquiries can be directed to the corresponding author.

## Abbreviations

The following abbreviations are used in this manuscript:

FRPs	Fiber-reinforced Polymers
BFRP	basalt fiber reinforcement polymer
CLTE	coefficient of linear thermal expansion
$T_{g}$	glass transition temperature
$\sigma_{t}$	tensile strength
$\sigma_{c}$	compressive strength
$\sigma_{b}$	bending strength
$M_{0}$	moisture equilibrium content
$D_{r}$	radial component of the diffusion coefficient
$D_{h}$	component of the diffusion coefficient along the reinforcement
$\frac{1}{1+\gamma /\beta }$	proportion of pseudo-equilibrium in the total equilibrium content of moisture
BFRP 6	basalt fiber reinforcement polymer bar with a nominal diameter 6 mm
BFRP 8	basalt fiber reinforcement polymer bar with a nominal diameter 8 mm
BFRP 10	basalt fiber reinforcement polymer bar with a nominal diameter 10 mm
BFRP 16	basalt fiber reinforcement polymer bar with a nominal diameter 16 mm
BFRP 20	basalt fiber reinforcement polymer bar with a nominal diameter 20 mm
